# Stable, multigenerational transmission of the bean seed microbiome despite abiotic stress

**DOI:** 10.1128/msystems.00951-24

**Published:** 2024-10-30

**Authors:** Abby Sulesky-Grieb, Marie Simonin, A. Fina Bintarti, Brice Marolleau, Matthieu Barret, Ashley Shade

**Affiliations:** 1 Department of Microbiology, Genetics and Immunology; Program in Ecology, Evolution and Behavior; and the Plant Resilience Institute, Michigan State University, East Lansing, Michigan, USA; 2 Univ Angers, Institut Agro, INRAE, IRHS, SFR QUASAV, F-49000 Angers, Beaucouzé, France; 3 Universite Claude Bernard Lyon 1, CNRS, INRAE, VeAgro Sup, Laboratoire d'Ecologie Microbienne LEM, CNRS UMR5557, INRAE UMR1418, Villeurbanne, France; University of Pretoria, Hatfield, South Africa

**Keywords:** drought, fertilizer, bacteria, high-throughput sequencing, 16S rRNA, legume, *Phaseolus vulgaris*, nutrients, endophyte

## Abstract

Microbiota that originate in the seed can have consequences for the education of the plant immune system, competitive exclusion of pathogens from the host tissue, and host access to critical nutrients. Our research objective was to investigate the consequences of the environmental conditions of the parent plant for bacterial seed microbiome assembly and transmission across plant generations. Using a fully factorial, three-generational experimental design, we investigated endophytic seed bacterial communities of common bean lines (*Phaseolus vulgaris* L.) grown in the growth chamber and exposed to either control conditions, drought, or excess nutrients at each generation. We applied 16S rRNA microbiome profiling to the seed endophytes and measured plant health outcomes. We discovered stable transmission of 22 bacterial members, regardless of the parental plant condition. This study shows the maintenance of bacterial members of the plant microbiome across generations, even under environmental stress. Overall, this work provides insights into the ability of plants to safeguard microbiome members, which has implications for crop microbiome management in the face of climate change.

**IMPORTANCE:**

Seed microbiomes initiate plant microbiome assembly and thus have critical implications for the healthy development and performance of crops. However, the consequences of environmental conditions of the parent plant for seed microbiome assembly and transmission are unknown, but this is critical information, given the intensifying stressors that crops face as the climate crisis accelerates. This study provides insights into the maintenance of plant microbiomes across generations, with implications for durable plant microbiome maintenance in agriculture on the changing planet.

## INTRODUCTION

The seed microbiome plays a role both in pathogen transmission and in shaping a plant’s beneficial microbiome (1, 2). Understanding the inheritance of microbiome members over plant generations, especially as mediated through seed transmission, is of interest for plant microbiome management and conservation (3–7). Plant microbiomes can be assembled via both horizontal transmission from the environment and vertical transmission from the parent plant through the seed microbiome (8–10). For seed transmission, microbial cells can either migrate through the vascular tissue or floral compartments and become packaged within the internal seed tissues or colonize seed surfaces (10–14). During germination, seed microbiome members that first colonize the plant can determine priority effects and drive the trajectory of microbiome assembly (15–17). Additionally, these “inherited” microbiome members enriched by the parent plant may provide important benefits for the offspring (18–21).

An unresolved question is how the environmental conditions of the parent plant influence seed microbiome assembly and whether stress can drive long-term alterations in the microbiome across generations (22–24). A few studies have found an influence of parental plant line on the resulting seed microbiome of the next generation (5, 25). Other studies have suggested an impact of parental stress on the seed microbiome after one generation, particularly drought in beans and wheat (26, 27), excess nutrients in beans (26), and salt stress in rice (28). A legacy effect of environmental stress on the seed microbiome could have consequences for the healthy assembly of the microbiome in the next plant generation (29).

**Fig 1:**
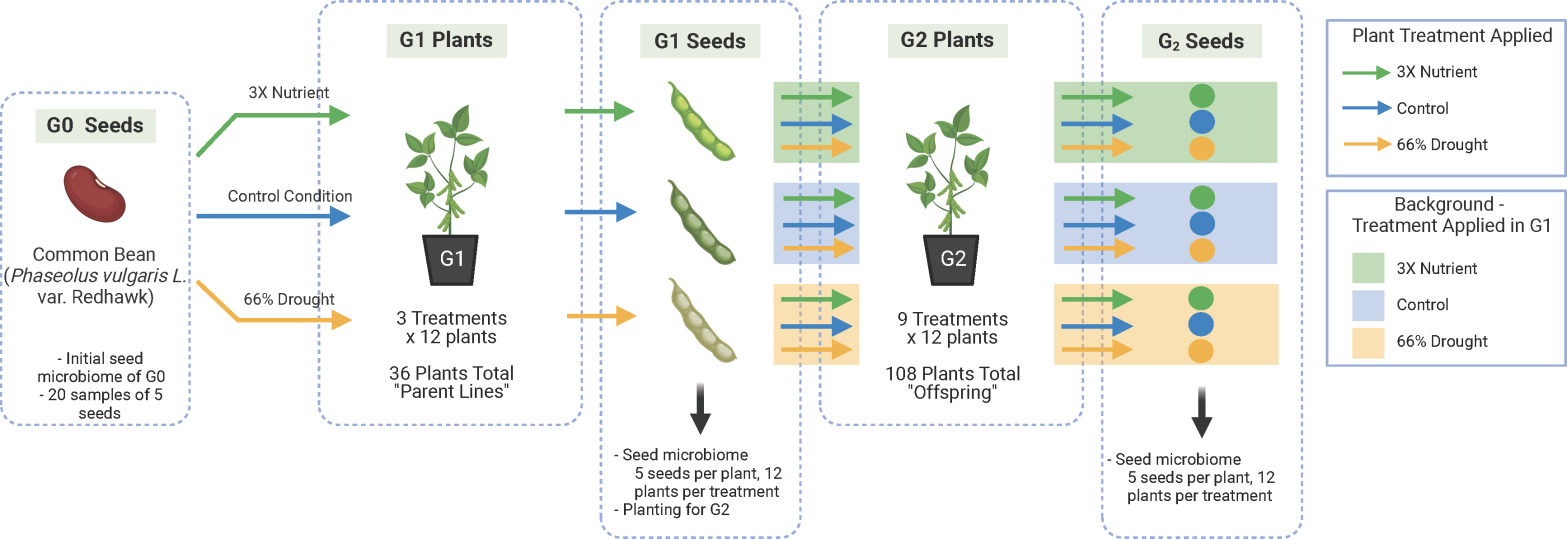
Experimental design. Seed microbiome samples were taken from the G0 seed pool, G1 plants, and G2 plants. We use “treatments” in the G1 plants to refer to the control condition (blue arrow), the nutrient excess (green arrow), and the drought (yellow arrow). We use “treatments” in the G2 plants to refer to every combination possible from the G1 lines in a factorial design. For example, one seed offspring from a control G1 parent line (background shade is blue) had a control condition in G2 (blue arrow in G2), another seed offspring from the same control G1 line had the excess nutrient condition (green arrow in G2), and another seed offspring from the same control G1 had drought condition (yellow arrow in G2), and the same was done for seeds from the parent lines for each G1 condition. In this way, each lineage and its experimental condition was tracked across the generations. Created with BioRender.com.

We conducted a multigenerational experiment in which we exposed common bean plants (*Phaseolus vulgaris* L.) to control conditions, drought or high nutrients during their early vegetative growth (Fig. 1). Plants were grown in agricultural soil in environmental chambers to allow for the natural assembly of their native microbiome while also controlling environmental conditions. *P. vulgaris* L. is a critical legume for global food security, supporting the health and livelihood of millions of people worldwide (30, 31). However, bean production is threatened by drought associated with warming and changes in precipitation patterns (32–34). Furthermore, beans grown in areas of high production are often managed with excess mineral fertilizer, which can impact the selection and stability of the plant microbiome (35) and also contribute to N_2_O emissions. Thus, a control condition plus two abiotic treatments, drought and nutrient excess, were selected because of their relevance to bean production in a changing climate.

We hypothesized that abiotic treatment of the parent plant characteristically alters the seed endophyte microbiome (26). We expected that each abiotic treatment would have specific consequences for the composition and stability of taxa transmitted across generations. We tested our hypothesis by planting a starting set of generation 0 (G0) bean seeds through two additional generations, exposed to either control growth conditions, drought, or excess nutrient treatments while tracking each plant’s parental line (Fig. 1). We applied standard 16S V4 rRNA gene amplicon sequencing of the seed bacterial endophyte communities for each of the three generations to assess the impact of the treatments on the seed microbiome and the potential for transmission of the bacterial taxa.

## MATERIALS AND METHODS

### Bean cultivar

*P. vulgaris* L. var. “Red Hawk” (36), developed by the Michigan State University Bean Breeding program, was selected as a common cultivar used in Michigan and one that is sensitive to drought. “Red Hawk” seeds were obtained from the Michigan State University Bean Breeding Program from their 2019 field harvest and stored at 4°C. Thus, their endophytic seed microbiome is the outcome of growth under agricultural field conditions. These seeds were designated as G0 and used to plant the first generation of the experiment. Their endophytic microbiomes were also analyzed as part of this study.

### Soil preparation

Agricultural field soil was collected for each planting group in September 2019, December 2020, May 2021, and September 2021 from a Michigan State University Agronomy Farm field that was growing common beans in 2019 (42°42'57.4"N, 84°27'58.9"W, East Lansing, MI, USA). The soil was analyzed to be a sandy loam with an average pH of 6.5 by the Michigan State University Soil, Plant, and Nutrient Laboratory.

When collecting the soil, we avoided the dry top 1–3 cm of soil that contained plant debris. Field soil was covered with air-tight lids and stored at 4°C until the experiment began. The soil was passed through a 4 mm sieve to remove rocks and plant debris and then mixed with autoclaved coarse vermiculite at a 50% vol/vol ratio immediately before planting.

### Surface sterilization and seed germination

Seeds were surface-sterilized with a solution of 10% bleach and 0.1% Tween20. Seeds were randomly selected from the bulk G0 seed supply or the harvested generation 1 (G1) seed supply, but we avoided seeds that were visibly cracked or moldy. Approximately 20 seeds were placed in a sterile 50-mL conical tube, 20–30 mL of bleach solution was added, and then, the seeds were soaked for 10 min, with agitation for 5 min. After soaking, the seeds were rinsed five times with sterile DI H_2_O. On the final rinse, 100 µL of rinse water was spread onto Tryptic Soy Agar (TSA) and Potato Dextrose Agar (PDA) plates to assess the efficacy of the seed surface sterilization. TSA plates were incubated overnight at 28°C and PDA plates at room temperature for 48 h. Seeds corresponding to plates that had microbial growth were discarded from the experiment and replaced with surface-sterile ones. For germination, the seeds were placed in a Petri dish lined with sterile filter paper and supplemented with 1–2 mL sterile DI H_2_O. Petri dishes were stored in the dark at room temperature for 3–4 days, with an additional 2 mL sterile DI H_2_O added after 2 days. Once seeds had sprouted radicle roots, they were transferred to soil.

### Growth conditions

For G1, three germinated seeds were planted into 3.78 L pots filled with the soil-vermiculite mixture and placed in a high-light BioChambers FLEX LED growth chamber with a 16 h day/8 h night cycle at 26°C and 22°C, respectively, and 50% relative humidity. Once seedlings emerged and reached the VC growth stage (vegetative growth with two cotyledons and primary leaves expanded), they were thinned to one seedling per pot. Plants were watered every other day with 300 mL 0.05% 15–10–30 water-soluble fertilizer solution, which then continued as the control condition (Masterblend International, Morris, IL, USA). At the V3 stage (vegetative growth with third trifoliate leaves expanded), treatments began for the drought- and nutrient-treated plants. Drought plants received 100 mL of 0.15% 15–10–30 fertilizer solution every other day, which was 66% less water than the control but an equivalent concentration of nutrients. Nutrient plants received 300 mL of 0.15% 15–10–30 fertilizer solution every other day, which was 3× concentrated nutrients than the control but with the same volume of water. After approximately 14 days of the treatment period, when plants reached the reproductive stage with the first open flowers, they were returned to the every-other-day water regime for the control plants until senescence. As plants began to dry, they were watered less frequently as needed. Mature seeds were collected from 12 plants per treatment for seed microbiome assessment and germination for the next generation.

For generation 2 (G2), seeds from the 36 G1 parental lines that received either control, drought, or nutrient conditions were grown under one of the three treatment conditions in G2 (Fig. 1). There were nine cross-generational treatment combinations total (G1_G2, *n* = 12 plants per treatment): Control_Control, Control_Drought, and Control_Nutrient; Drought_Control, Drought_Drought, and Drought_Nutrient; and Nutrient_Control, Nutrient_Drought, and Nutrient_Nutrient. Six seeds from each parental line were surface-sterilized and germinated as described above, then planted in the field soil-vermiculite mixture in seedling trays in the growth chamber under the conditions stated above. G2 plants were grown in three randomized planting groups, with each planting group containing parental lines from all three treatments. Once plants reached the VC stage, three healthy seedlings per parent line were transferred to 3.78 L pots. Each G1 parental line provided one seed and thus one offspring per G2 treatment for a total of 108 plants in G2. Plants were watered according to the conditions and treatment timeline in G1.

### Plant and seed harvest

Once the plants had senesced and pods were dried, plant compartments were harvested for microbiome analysis. Seed pods were removed and stored in a sterile Whirl-pak bag. Root systems were removed from the pot and shaken to remove loose soil. Roots were collected in a Whirl-pak bag, and associated rhizosphere soil was collected in a sterile 50 mL conical tube. Pods and seeds per plant were counted, and seeds were removed from the pods and stored at 4°C until planting or processing. Roots and rhizosphere soil were stored at −80°C until further analysis. Five seeds per plant were selected for microbiome analysis and stored in a 15 mL conical tube at −80°C until DNA extraction was performed.

### DNA extractions

We adhered to general standards in the field regarding the handling and analysis of low microbial biomass samples assessed with high-throughput sequencing (37). DNA extractions were performed on sets of five randomly selected seeds per plant, which is our unit of microbiome sampling. For the G0 bulk seed, 20 sets of five randomly selected seeds from each plant were analyzed (these five seeds did not necessarily come from the same parent plant). Seeds were analyzed from 36 parent plants for G1 and from 108 offspring for G2. Seeds were thawed and surface-sterilized according to the method above, and then, microbial DNA was extracted from the endophytic compartment using a protocol adapted from (8, 38). Following surface sterilization, the seeds were sliced in half lengthwise along the natural division of the cotyledons with a sterile razor blade. Sliced seeds were placed in a 50-mL conical tube, and 20–30 mL of sterile phosphate-buffered saline (PBS) with 0.05% Tween 20 was added. Seeds were soaked overnight at 4°C with constant agitation on a surface shaker at 160 rpm. After soaking, tubes were centrifuged at 4500 × *g*, 4°C for 1 hour. Seed tissue and supernatant were removed, and the remaining pellet was transferred to a 1.5 mL microcentrifuge tube. Pellets were stored at −80°C until extraction with the E.Z.N.A. Bacterial DNA kit (Omega Bio-tek, Inc., Norcross, GA, USA) following the manufacturer’s protocol with the following modifications. The seed material pellet was resuspended in 100 µL TE buffer, 10 µL kit-provided lysozyme was added, and the samples were vortexed thoroughly and incubated at 37°C for 1 h. The glass bead step from the E.Z.N.A. kit was utilized with 25–30 mg glass beads provided, and samples were vortexed at maximum speed for 10 min in a 24-tube vortex adapter. After adding the Proteinase K, the samples were incubated in a shaking heat block at 55°C for 2 h. In the final step, DNA was eluted in 60 µL elution buffer and incubated at 65°C for 10 min before centrifuging into the final tube.

DNA extractions were performed in randomized batches within each generation (Table S2). For each batch, negative and positive extraction controls were included. The negative control was 3 mL sterile PBS + Tween buffer, and the positive control was an aliquot of a mixture of cells from a custom-made mock bacterial community in 3 mL buffer (39). These controls were soaked overnight alongside the seed samples and then processed and sequenced as described for the seeds, and then ultimately used to perform batch-informed bioinformatic sequence decontamination (40).

The root samples were thawed at room temperature, and a 1–2 inch section of the main root system was cut and used for root DNA extraction, which combined the rhizoplane and endophytic bacteria inside the root tissues. The selected sections were briefly rinsed in sterile DI water and then placed in a clean mortar. Liquid nitrogen was added to the mortar, and the roots were finely ground with a pestle and used for DNA extraction with the DNeasy PowerSoil Pro DNA Kit (Qiagen, Germantown, MD, USA) following the manufacturer’s instructions with the following modifications. In step one, 750 µL solution CD1 was used with 50 µL ATL buffer (Qiagen, Germantown, MD, USA). The bead beating step was performed for 15 min on a vortex genie 24-tube adapter at maximum speed. Finally, 60 µL of the final elution buffer C6 was used, and the tubes were incubated for 10 min before centrifugation.

Rhizosphere soil was thawed at room temperature, and DNA was extracted with the DNeasy PowerSoil Pro DNA Kit with the same ATL buffer variation as the roots, above. Negative controls were included with each batch of DNA extractions from the start of the extraction procedure, and one positive mock community control was included with each compartment sample set (roots or rhizosphere) (39). Control extraction samples were sequenced with the experimental samples for use in data processing.

### Amplicon sequencing

Sequencing of the V4 region of the 16S rRNA gene (515 F-806R) (41, 42) was performed at the Environmental Sample Preparation and Sequencing Facility (ESPSF) at Argonne National Laboratory (Lemont, IL, USA). Twelve negative controls of blank library preparations were included and used for bioinformatic decontamination as well as manual inspection for potential contaminants. The DNA was PCR-amplified with region-specific primers that include sequencer adapter sequences used in the Illumina Nextseq2K flowcell; FWD:GTGYCAGCMGCCGCGGTAA; REV:GGACTACNVGGGTWTCTAAT (41–45). Each 25 µL PCR reaction contained 9.5 µL of MO BIO PCR Water (Certified DNA-Free), 12.5 µL of QuantaBio’s AccuStart II PCR ToughMix (2× concentration, 1× final), 1 µL Golay barcode tagged forward primer (5 µM concentration, 200 pM final), 1 µL reverse primer (5 µM concentration, 200 pM final), and 1 µL of template DNA. The conditions for PCR were as follows: 94°C for 3 min to denature the DNA, with 35 cycles at 94°C for 45 s, 50°C for 60 s, and 72°C for 90 s, with a final extension of 10 min at 72°C to ensure complete amplification. Amplicons were then quantified using PicoGreen (Invitrogen) and a plate reader (Infinite 200 PRO, Tecan). Once quantified, the volumes of each of the products were pooled into a single tube in equimolar amounts. This pool was then cleaned up using AMPure XP Beads (Beckman Coulter) and then quantified using a fluorometer (Qubit, Invitrogen). After quantification, the pool was diluted to 2 nM, denatured, and then diluted to a final concentration of 6.75 pM with a 10% PhiX spike for sequencing. Amplicons were sequenced on a 251bp × 2 bp NextSeq2000.

### Sequence data processing

Fastq files were processed in QIIME2 after primer removal by the sequencing center (QIIME2 version: 2022.8.0)(46). Sample fastq files were imported to QIIME2 format, and samples were denoised, truncated, and merged using DADA2 with a forward truncation length of 191 and reverse truncation length of 84 (47). Amplicon sequence variants (ASVs) were defined at 100% sequence identity, and 16S taxonomy was assigned with the Silva database release 138 at the default confidence value of 0.7, and taxonomy and ASV tables were exported for further analysis in R (48).

Data analyses were performed in R version 4.3.1 and R Studio version 2023.06.1+524 (49). There were 126.8 million merged DNA reads prior to host removal and decontamination. ASV, taxonomy, metadata tables, and phylogenetic tree files were imported into the phyloseq package, and host reads classified as chloroplast and mitochondria were removed using the subset_taxa() command in the phyloseq package version 1.44.0 (50). Ninety percent of the total DNA reads and 13% of the ASVs were removed as the host reads, leaving 12.3 million total bacterial DNA reads. Host-filtered data sets were next decontaminated with the decontam package version 1.20.0 at the 0.1 threshold using the negative and positive controls from each extraction group (51). Finally, we manually inspected the negative controls from the library preparation and found one potential ASV contaminant that was not automatically flagged by our decontam procedure. Reads associated with this ASV were present in abundances of 3,000–120,000 reads in all the negative controls for the library preparation provided by the sequencing center and consistently in all but one of our true samples. This ASV, identified taxonomically as a *Porphyromonas*, has had close relatives previously reported associated with plants, particularly to the endophytic compartment of legume root nodules (52). However, out of an abundance of caution, we manually removed it as a putative contaminant from further analyses with the phyloseq package. After decontamination and manual removal of the putative contaminating ASV, there were 398,365 total DNA reads with a range of 381–5423 reads per sample in the full data set (Fig. S1).

Rarefaction curves were created using the rarecurve() command in the vegan package version 2.6–4 (53) (Fig. S1). Data sets were then subset for further analysis using the ps_filter() command in the microViz package version 0.10.10 (54). Since seed microbiomes typically have low bacterial diversity containing tens to hundreds of taxa, and vertical transmission of specific ASVs was a primary area of investigation in this study, the full data set was preserved to ensure full observation ASVs (38).

### Ecological statistical analysis

Alpha diversity was assessed in R using estimate_richness() in phyloseq with an ANOVA, and figures were created using the plot_richness() command from phyloseq with the ggplot2 package version 3.4.2 (55). Faith’s phylogenetic diversity was calculated with calculatePD() from the biomeUtils package version 0.022 (56). ANOVAs were performed with the base R stats command aov(). Weighted UniFrac distances were calculated with distance() in phyloseq and used for all analyses of beta diversity, and PERMANOVA statistical tests were performed with adonis2() from the vegan package. We used Weighted UniFrac distance because it explained the most microbiome variation relative to other resemblances we also considered (e.g., Bray-Curtis, Jaccard, etc). Post-hoc analysis on the PERMANOVA results was performed with pairwise.adonis2() from pairwise Adonis version 0.4.1 (57). Beta dispersion was assessed with the betadisper() and permutest() commands from the vegan package. We created an “upset plot” with the UpSetR package version 1.4.0 (58), and statistical analyses were performed with leveneTest() from the car package (59) and kruskal_test() and dunn_test() from the rstatix package (60). Beta diversity ordinations were created with ordinate() from phyloseq with ggplot2. Additional data analysis was performed in the tidyverse package version 2.0.0 (61) and dplyr package version 1.1.2 (62). Amplicon sequence variant (ASV) transmission was analyzed as count data, and Pearson’s chi-squared tests were performed with chisq.test() from the base R stats package. Seed core microbiota identified in (63) were compared with transmitted ASVs, and Venn diagrams were produced with the VennDiagram package version 1.7.3 (64). To compare the prevalent ASVs in this study to the 48 core bean rhizosphere microbiome taxa identified by (65, 65), the fasta sequences for each core OTU were used as a query set in a two-sequence nucleotide BLAST on the National Center for Biotechnology Information (NCBI) database website, and the fasta sequences from the seed ASVs were compared with the 48 core taxa at least 97% identity (66).

## RESULTS AND DISCUSSION

**Fig 2:**
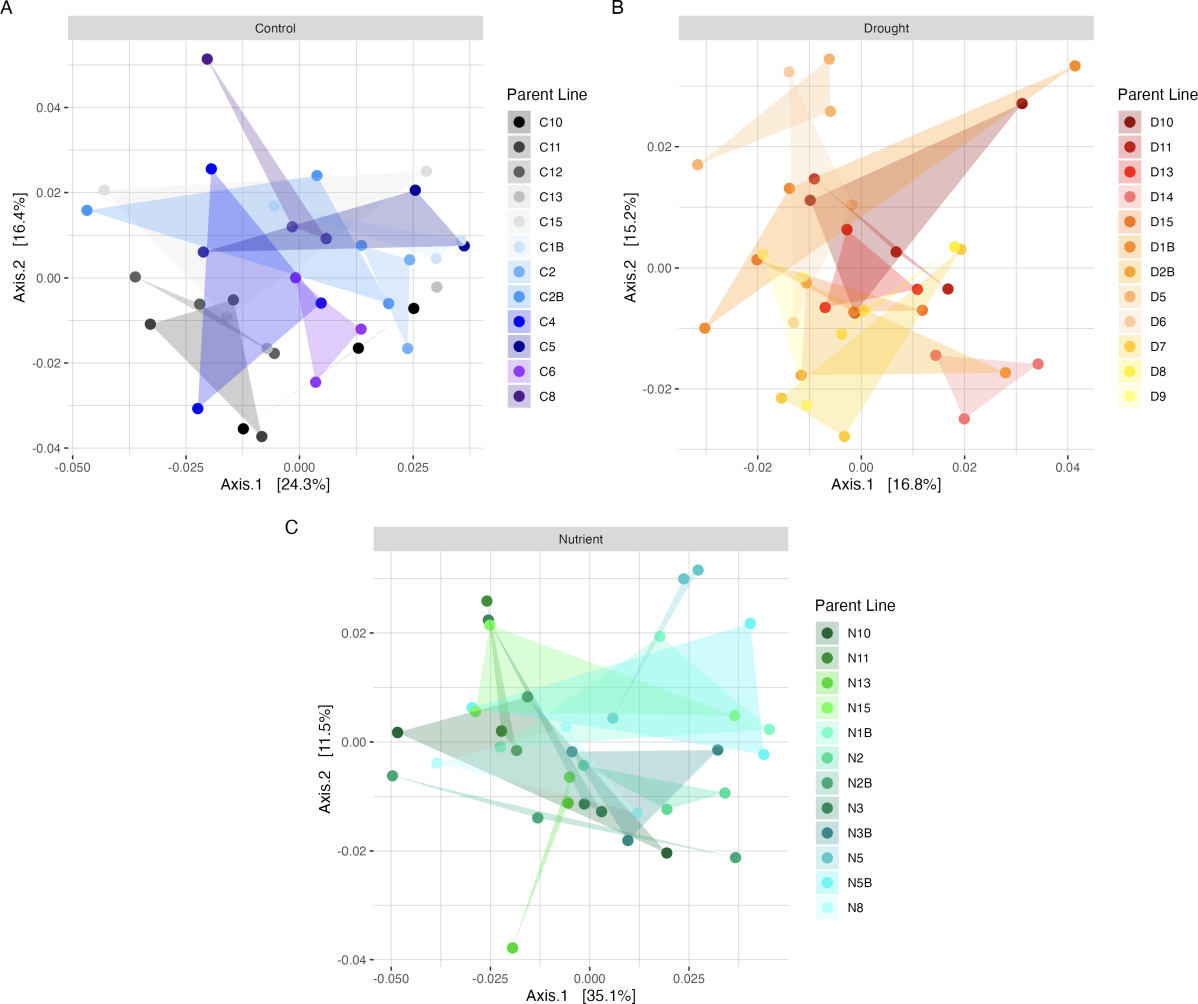
PCoA plots of weighted UniFrac beta diversity in G2, subset by the parent plant treatment in G1. When plant lines are grouped by G1 parent treatment, the parent plant line is not significant in the control and nutrient lines (See Table S2) but is statistically supported in the drought lines (PERMANOVA, Drought: r2 = 0.39, F = 1.41, *P* = 0.003). Bars above the ordinations indicated the treatment applied to the parent plants in G1: A, control; B, drought; and C, nutrient. The three offspring from each parent are connected by triangles to aid in visualization. Sample G2_9, the line C13 nutrient offspring, was removed from Figure A as an outlier. However, statistics were performed with this sample included.

The drought and nutrient treatments did not influence seed microbiome alpha or beta diversity in either generation (Fig. S2 and S3; Table S2). Furthermore, we detected no legacy influence of the G1 treatments on the G2 seed microbiomes and no overarching differences in the microbiomes across the G1 and G2 generations (Fig. S3; Table S2). However, there was an appreciable influence of parental line for those G2 plants that originated from the G1 drought treatment (Fig. 2, PERMANOVA *post-hoc* r^2^ = 0.39 F = 1.41 *P* = 0.003 for panel B), but not for the control or nutrient parental lines.

**Fig 3:**
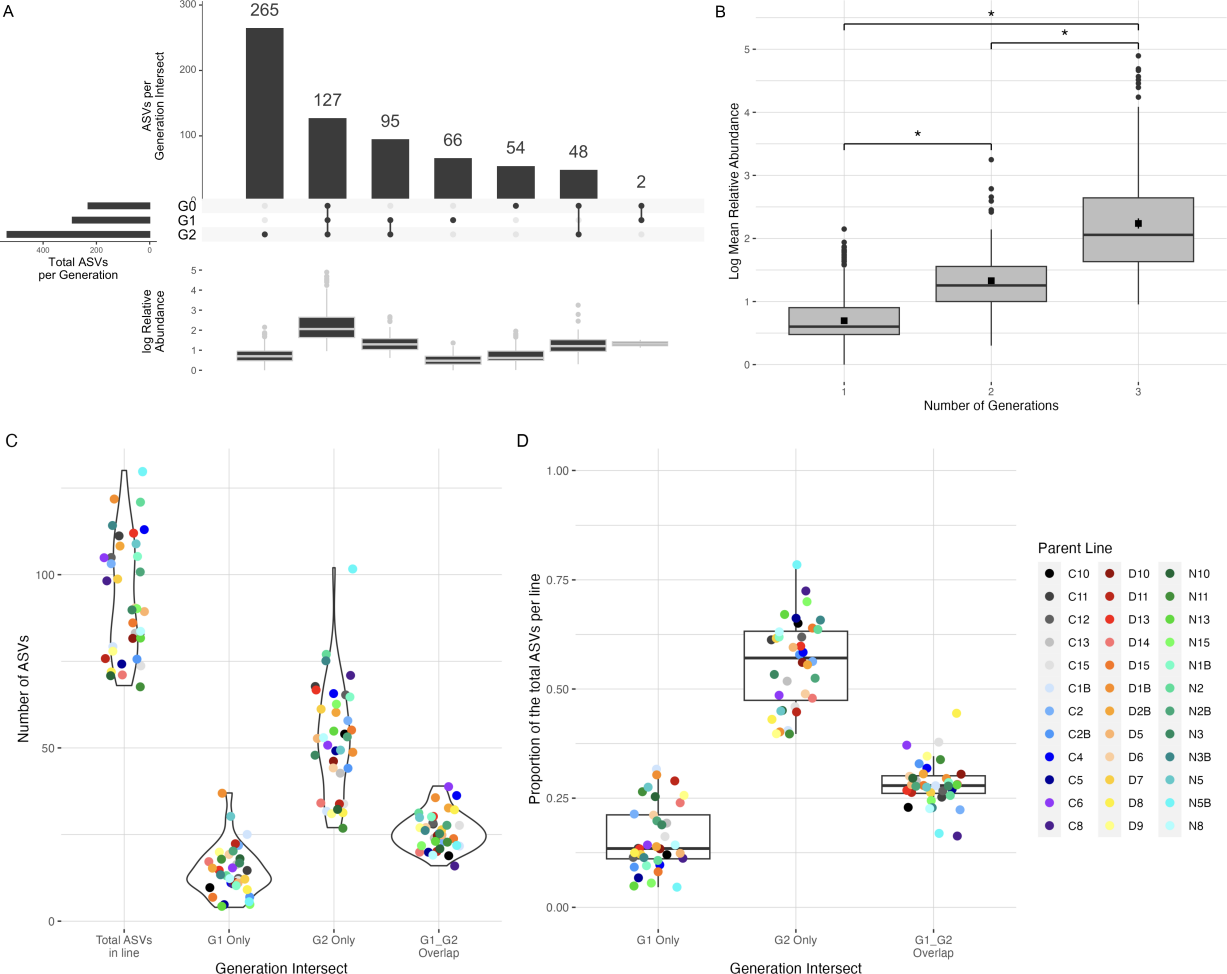
Unique and overlapping ASVs between generations. A, Number of ASVs and relative abundance of ASVs per generation intersect across all samples. G0 *n* = 20 samples (five seeds per sample), G1 *n* = 36, G2 *n* = 108. B, Log relative abundance of ASVs found in one, two, or three generations. Of 657 total ASVs detected, ASVs found in all three generations are significantly more abundant in the data set than ASVs found in only one or two generations. Black squares indicate the mean value. (Kruskal-Wallis test: test-statistic = 361.96, df = 2, *P* < 0.0001; post-hoc Dunn’s test with Benjamini-Hochberg correction, 1 vs 3 generations: test-statistic = 17.71, adjusted *P* < 0.0001, 2 vs 3 generations: test-statistic = 5.84, adjusted *P* < 0.0001). C, Total number of ASVs per parent line and number of ASVs found in G1, G2, or overlapping. “G1_G2 Overlap” is defined as ASVs present in both the G1 sample and at least one G2 offspring within a parent line. Ninety-eight ASVs were identified as overlapping within parent lines, and there were overlapping ASVs identified in all 36 lines. D, Proportion of the total ASVs per line found in G1, G2, or overlapping. Boxplots represent the median values and first and third quartiles, and whiskers represent the 95% CI. C, D, and N in parent line IDs denote lines that received control, drought, or nutrient treatment in G1, respectively.

Instead, we found evidence of stable transmission of 127 of 657 detected amplicon sequence variants (ASVs) across all three generations, regardless of the experimental treatment (Fig. 3A). The 127 ASVs detected in all three generations were in higher relative abundance than the ASVs that were only seen in one or two generations (Kruskal-Wallis test: test-statistic = 361.96, df = 2, *P* < 0.0001; post-hoc Dunn’s test with Benjamini-Hochberg correction: 1 v. three generations: test-statistic = 17.71, adjusted *P* < 0.0001, 2 v. three generations: test-statistic = 5.84, adjusted *P* < 0.0001) (Fig. 3B). Furthermore, the genus-level taxonomic profiles of G0, G1, and G2 microbiomes were highly comparable (Fig. S4), suggesting a consistent taxonomic signature of the ASVs detected across seed generations.

We identified ASVs overlapping between a G1 parent plant and at least one of its offspring in G2, which we call “overlapping ASVs.” There were 98 overlapping ASVs discovered among the 36 lines, 69 of which were found in at least two parent lines and 42 of which were common to all treatments. The average proportion of overlapping ASVs in each line was 28% and ranged from 16% to 44% of the total ASVs detected (Fig. 3C and D).

**Fig 4:**
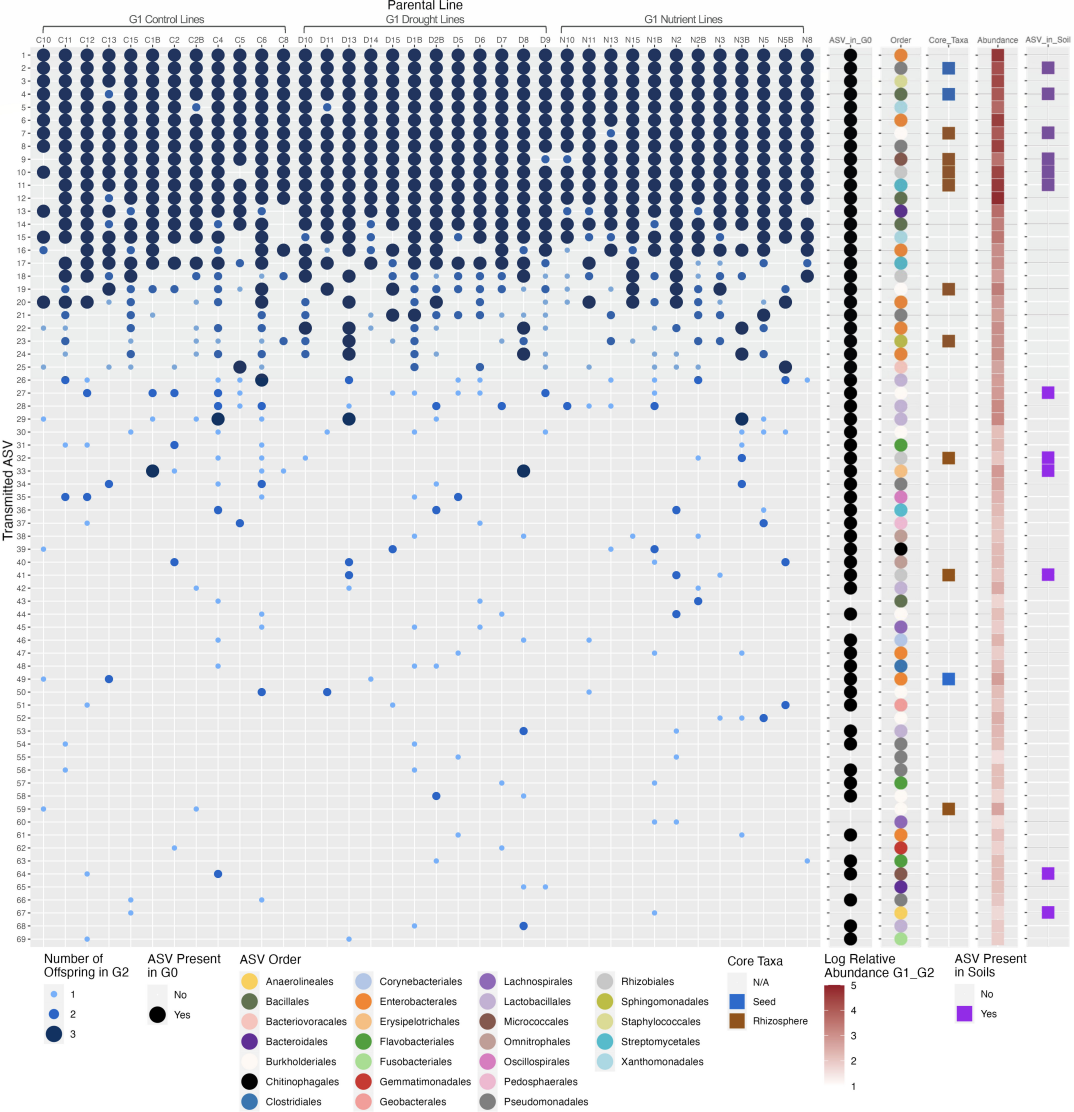
Prevalence in lines, transmission, core taxa identity, and relative abundance of overlapping ASVs in G2 offspring. Of the 98 ASVs that were found overlapping between G1 and G2 within parent lines, 29 ASVs that were only in one parent line were removed, and the remaining 69 ASVs are listed above, ordered from presence in the highest number of lines to lowest number of lines. Blue dots represent the number of G2 offspring containing the ASV in each line. There was no significant difference in ASV transmission in G2 offspring between G1 treatments or parental lines (Pearson’s chi-squared test, see Table S2 for details). Sixty of these ASVs are also found in the G0 data set indicated by black dots. The taxonomy of each ASV identified at the order level is indicated by colored dots in the order column (26 orders). Blue and brown squares in the Core_Taxa column indicate identity with seed or rhizosphere core taxa, respectively. Red boxes in the abundance column represent the log relative abundance of the ASV overall across G1 and G2. Purple squares in the right-most column indicate ASVs that were also found in the root, rhizosphere, and/or source soil samples in this experiment.

Eight overlapping ASVs were present in all 36 parental lines, and an additional 13 ASVs were found in at least half of all parental lines. These 21 ASVs generally had 100% transmission to all three G2 offspring per parent line (Fig. 4). At the same time, we noticed that less prevalent ASVs were not consistently transmitted in all offspring within lines. The G1 treatment did not impact the average transmission of the ASVs in the G2 offspring, and there was no significant difference in average transmission between parental lines (Pearson’s chi-squared test, see Table S2) (Fig. 4).

Most overlapping ASVs that were found in at least two parental lines were also detected in the G0 microbiomes (60 of 69 ASVs) (Fig. 4). These ASVs were taxonomically diverse and included 39 families and 26 orders. Many overlapping ASVs were also highly abundant (Fig. 4). Their genus-level taxonomic compositions in G2 were very similar within and across parent lines (Fig. S5).

To seek generalities with other relevant studies, we compared the 69 overlapping ASVs detected in this study with the six “core” common bean seed microbiome ASVs identified by (63) and with the 48 “core” common bean rhizosphere OTUs identified by (65). These previously reported core taxa were identified in a meta-analysis of multiple studies that used amplicon sequencing to assess seed-associated microbiomes and are hypothesized to be important for health in common beans. Three core common bean seed ASVs (63) were found in the overlapping G1-G2 data set, specifically from the *Pseudomonas*, *Bacillus*, and *Pantoea* genera (Table 1; Fig. 4; Fig. S6). Two ASVs, the *Pseudomonas* and *Bacillus* core members, were detected in all 36 parental lines, whereas the *Pantoea* core seed microbiome member was found in only three parental lines (Table 1). There were nine ASVs aligned at 97% identity to the previously identified core rhizosphere taxa (65) (Table 1; Table S3; Fig. 4). These ASVs were classified in the families Comamonadaceae, Devosiaceae (genus *Devosia*), Methyloligellaceae, Oxalobacteraceae (genus *Massilia*), Rhizobiaceae (genus *Ochrobactrum*), Sphingomonadaceae (genus *Sphingomonas*), Streptomycetaceae (genus *Streptomyces*), and Micrococcaceae in the genus *Arthrobacter*, of which our seed ASV aligned at 98.8% identity to the most abundant core OTU in the rhizosphere study (Table 1; Table S3). These genera are commonly associated with plant microbiomes (67–71). Furthermore, many of these detected taxa or their close relatives have been previously cultivated by us from the bean seed endophyte (72, 73), suggesting that they are viable members and not simply persistent DNA.

**TABLE 1:** Stable ASVs that were detected in all three generations^*a*^

ASV	Lines	Core taxa	Class	Order	Family	Genus	Detection in other compartments
1	All lines	-	Gammaproteobacteria	Enterobacterales	Enterobacteriaceae		-
2	All lines	S	Gammaproteobacteria	Pseudomonadales	Pseudomonadaceae	*Pseudomonas*	Soil
3	All lines	-	Bacilli	Staphylococcales	Staphylococcaceae	*Staphylococcus*	-
4	All lines	S	Bacilli	Bacillales	Bacillaceae	*Bacillus*	Root, Rhizo, Soil
5	All lines	-	Gammaproteobacteria	Xanthomonadales	Xanthomonadaceae	*Stenotrophomonas*	-
6	All lines	-	Gammaproteobacteria	Enterobacterales	Enterobacteriaceae	*Escherichia-Shigella*	-
7	All lines	R	Gammaproteobacteria	Burkholderiales	Oxalobacteraceae	*Massilia*	Root
8	All lines	-	Gammaproteobacteria	Pseudomonadales	Pseudomonadaceae	*Pseudomonas*	-
9	35 lines	R	Actinobacteria	Micrococcales	Micrococcaceae	*Arthrobacter*	Rhizo
10	35 lines	R	Alphaproteobacteria	Rhizobiales	Rhizobiaceae	*Ochrobactrum*	Root, Rhizo, Soil
11	35 lines	R	Actinobacteria	Streptomycetales	Streptomycetaceae	*Streptomyces*	Rhizo
12	35 lines	-	Bacilli	Bacillales	Bacillaceae	*Bacillus*	-
13	34 lines	-	Bacteroidia	Bacteroidales	Bacteroidaceae	*Bacteroides*	-
14	34 lines	-	Bacilli	Bacillales	Bacillaceae	*Bacillus*	-
15	32 lines	-	Gammaproteobacteria	Xanthomonadales	Xanthomonadaceae	*Xanthomonas*	-
16	28 lines	-	Gammaproteobacteria	Enterobacterales	Enterobacteriaceae		-
17	28 lines	-	Actinobacteria	Streptomycetales	Streptomycetaceae	*Streptomyces*	-
18	27 lines	-	Alphaproteobacteria	Rhizobiales	Rhizobiaceae	*Ochrobactrum*	-
19	24 lines	R	Gammaproteobacteria	Burkholderiales	Comamonadaceae		-
20	23 lines	-	Gammaproteobacteria	Enterobacterales	Enterobacteriaceae	*Escherichia-Shigella*	-
21	18 lines	-	Gammaproteobacteria	Pseudomonadales	Pseudomonadaceae	*Pseudomonas*	-
49	three lines	S	Gammaproteobacteria	Enterobacterales	Erwiniaceae	*Pantoea*	-

^*a*^ Eight ASVs are found in all 36 parental lines, two of which are core seed microbiome taxa (reported by (63), labeled “S”). Thirteen additional ASVs were present in 50% or more of the parental lines. An additional core seed microbiome member was found in three parental lines. Five of these ASVs align to bean rhizosphere core OTUs at >97% identity (as reported by (65), labeled “R”). All ASVs listed were also found in generation 0 seeds. Six taxa were also found in the associated plant soil environment: “Soil” indicates that the ASV was detected in the source field soil in which the plants were planted, “root” indicates that the ASV was detected in the samples including root surface and endophytic tissue, and “rhizo” indicates that the ASV was detected in the rhizosphere soil of the plants used in the experiment.

Our multigenerational study indicates that numerous seed microbiome members were consistently packaged in the seed endophytes of the G0, G1, and G2 plants, regardless of the growth conditions (field as in G0 or environmental chamber as in G1 and G2) or abiotic treatment applied to the plants. This suggests a consistent and stable seed microbiome transmission for common beans. This is an unexpected result because, for many plant species, the seed microbiome has been reported to have relatively low diversity (tens to hundreds of taxa), low biomass/small community size (dozens of cells), and notably high compositional variability (4, 38, 63), suggesting an influence of stochasticity in the assembly. Our results indicate that against a background of high variability, a handful of members of the seed endophyte microbiome exhibit stable transmission. These transmitted taxa could not have been identified before because previous studies have focused on only one generation, and, perhaps more importantly, on seeds that were pooled across multiple parent plants so that the lineage information was lost and could not be used for interpretation. These stable seed microbiome taxa may establish mutualistic or commensal associations and persist through the plant life cycle until packaged within the seed for the next generation. It also suggests that a parental effect on the seed microbiome has the potential to outweigh abiotic effects. More research is needed to directly test these next hypotheses.

Our results cannot address the exact mode of transmission of the seed endophyte microbiome, which is a limitation of the work. It remains unclear whether the seed endophytes are passed directly via vertical transmission [e.g., from seed to plant to seed via the vascular tissue as previously reported as the primary pathway for common bean (11, 74)] or re-acquisition (e.g., parent plants selectively recruit the same taxa from the environment). However, our data show that many of these taxa were not detected in the associated rhizosphere, root, and source soil samples from this experiment (Fig. 4; Fig. S7; Table 1), suggesting that vertical transmission is likely for at least some of the prevalent endophytic seed taxa. Regardless of the transmission mode, the seed microbiome members’ stability despite abiotic treatment, their prevalence and high relative abundance, and their consistent detection across several bean microbiome investigations (63, 65) suggest a strong selection of the plant seed environment for these taxa. Thus, although our initial hypothesis about the importance of abiotic treatment in driving seed endophyte microbiome variation was not supported, there was clear evidence that a stable seed microbiome was transmitted in all conditions of our study.

As the need for sustainable solutions to maintain or improve agricultural productivity increases, plant microbiome management, microbiome engineering, and breeding plants for improved microbiomes will be critical strategies (75–77). Applying beneficial plant microbiome members via seed treatments or soil inoculation has shown promise in improving plant growth or health (78, 79). Our objectives here were to understand how the environmental conditions of the parent plant impact the assembly and transmission of the seed endophyte microbiome. The stably transmitted bean seed microbiome members identified provide targets for future research to understand how to shape legume microbiomes to improve crop yield, health, and resilience (80, 81).

## Data Availability

Data analysis code can be found at (https://github.com/ShadeLab/Seed_transmission_Common_Bean). Raw sequences, including from controls, can be found on the NCBI Sequence Read Archive under BioProject number PRJNA1058980.

## SUPPLEMENTAL MATERIAL

Supplemental material msystems.00951-24-s0001.pdf Figures S1-S7, captions for Tables S1 and S3, and Table S2.

Table S1 msystems.00951-24-s0002.xlsx MIMARKS compliant contextual information for each sample (e.g., plant treatment data, extraction batches).

Table S3 msystems.00951-24-s0003.xlsx List of 69 ASVs from Figure 2 with taxonomic identification, core taxa membership and % identity, and 16S V4 sequence.

ASM does not own the copyrights to Supplemental Material that may be linked to, or accessed through, an article. The authors have granted ASM a non-exclusive, world-wide license to publish the Supplemental Material files. Please contact the corresponding author directly for reuse.
