# Supplementary material for: Stable, multigenerational transmission of the bean seed microbiome despite abiotic stress
Source: mSystems. 2024 Oct 30;9(11):e00951-24. doi: 10.1128/msystems.00951-24 (PMC11575401; doi:10.1128/msystems.00951-24)
Supplement: Supplemental material — Figures S1-S7, captions for Tables S1 and S3, and Table S2. [file msystems.00951-24-s0001.pdf]

Supplemental Materials for

Stable, multigenerational transmission of the bean seed microbiome despite abiotic stress

Abby Sulesky-Grieb[1], Marie Simonin[2], A. Fina Bintarti[3], Brice Marolleau[2], Matthieu Barret[2], Ashley Shade[4*]

Supplemental Figures and Tables

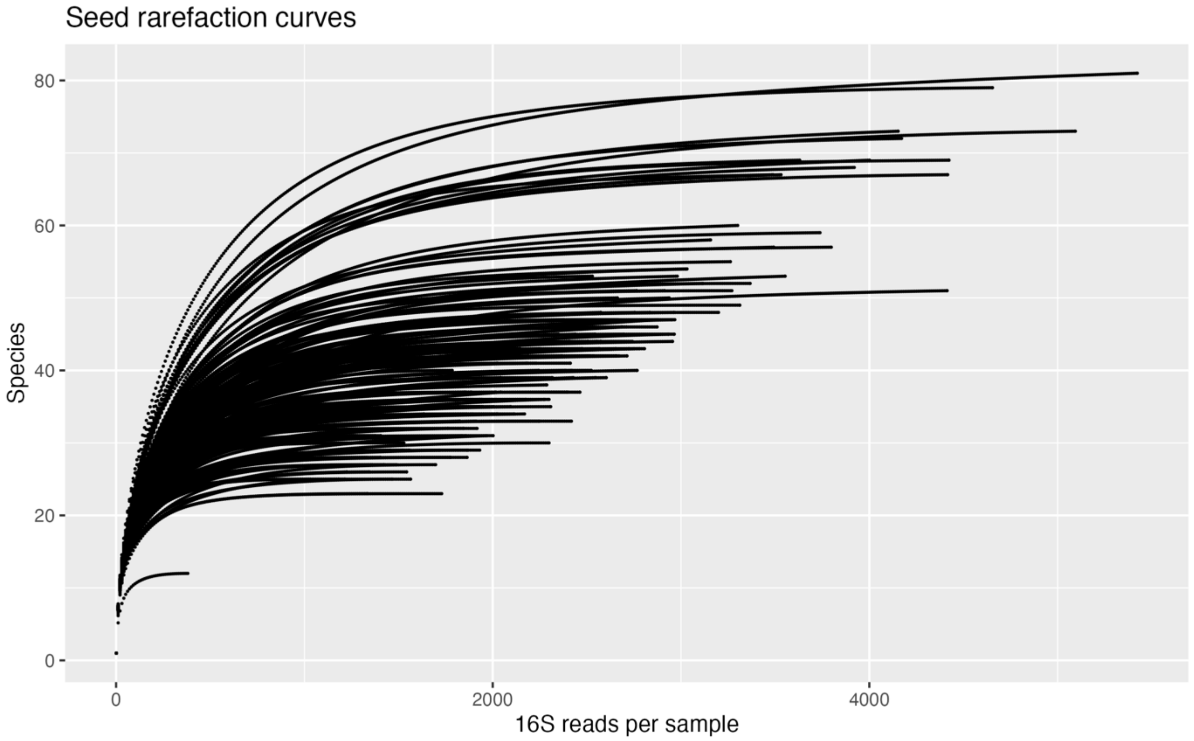

Fig. S1. Rarefaction curves of quality-filtered microbiome profiles (host reads removed, see methods). Each line represents one seed microbiome sample (pool of 5 seeds from the same parent plant). The DNA read range is 381-5423. All samples reached a plateau indicating a sufficient coverage to characterize seed microbiome diversity.

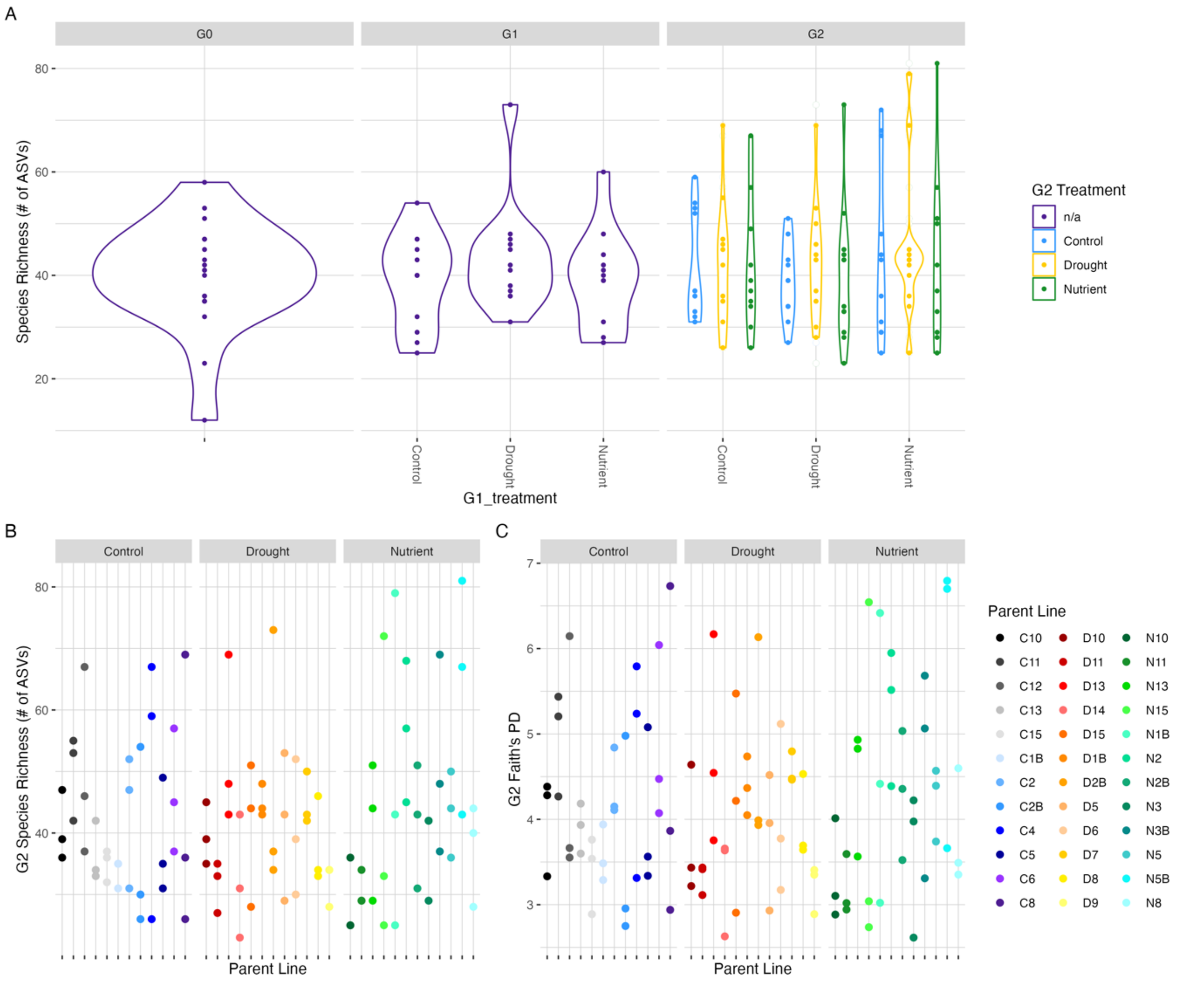

Fig. S2. Alpha Diversity. (A) Number of ASVs observed in each of the seed microbiome samples across all generations. Five seeds were used for each sample. G0 n=20 samples, G1 and G2 n=12 samples per treatment group. There is no influence of treatment groups on the species richness observed in either G1 or G2 (ANOVA, G1_treatment: F= 0.150, p-value=0.861. G1_G2: F=0.393, p-value=0.923). **(B)** Number of ASVs observed, and **(C)** Faith's Phylogenetic Diversity of G2 seed samples by parental line. Gray bars indicate treatment applied to the parent plant in G1. There are no significant differences between parent lines in either Richness or PD measure (ANOVA, Richness: F=1.122, p=0.334; PD: F=1.123, p=0.332).

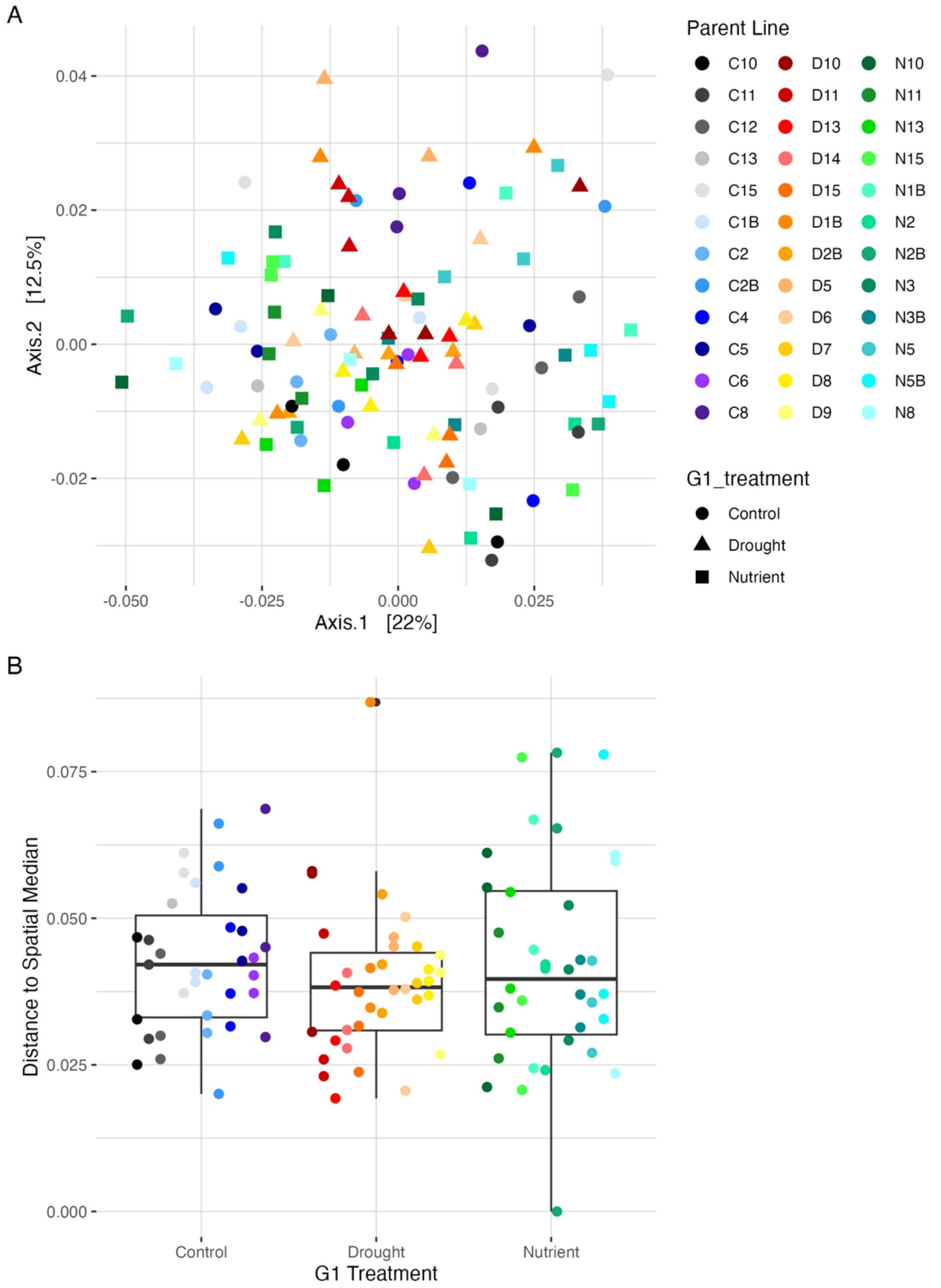

Fig. S3. Beta diversity in Generation 2 seed samples. (A) PCoA of Weighted Unifrac distance of G2 seed samples. Points represent three offspring from each parent line, each of which received a different treatment in G2. Parent plant line is the only significant explanatory variable in the G2 samples (PERMANOVA, r^2=0.35582, F = 1.22276, p=0.0042**). **(B)** Beta dispersion around the spatial median of Weighted Unifrac distances in G2 seed samples. Lines are grouped by G1 parent treatment, represented by black boxplots. G1 treatment and parent line are not significant. (ANOVA, G1 Treatment: DF=2, F-value= 1.4246, p= 0.2132. Line: DF: 35, F-value=1.0388, p=0.4541). Sample G2_9, the line C13 Nutrient offspring, was removed from the figures as an outlier. However, statistics were performed with this sample included.

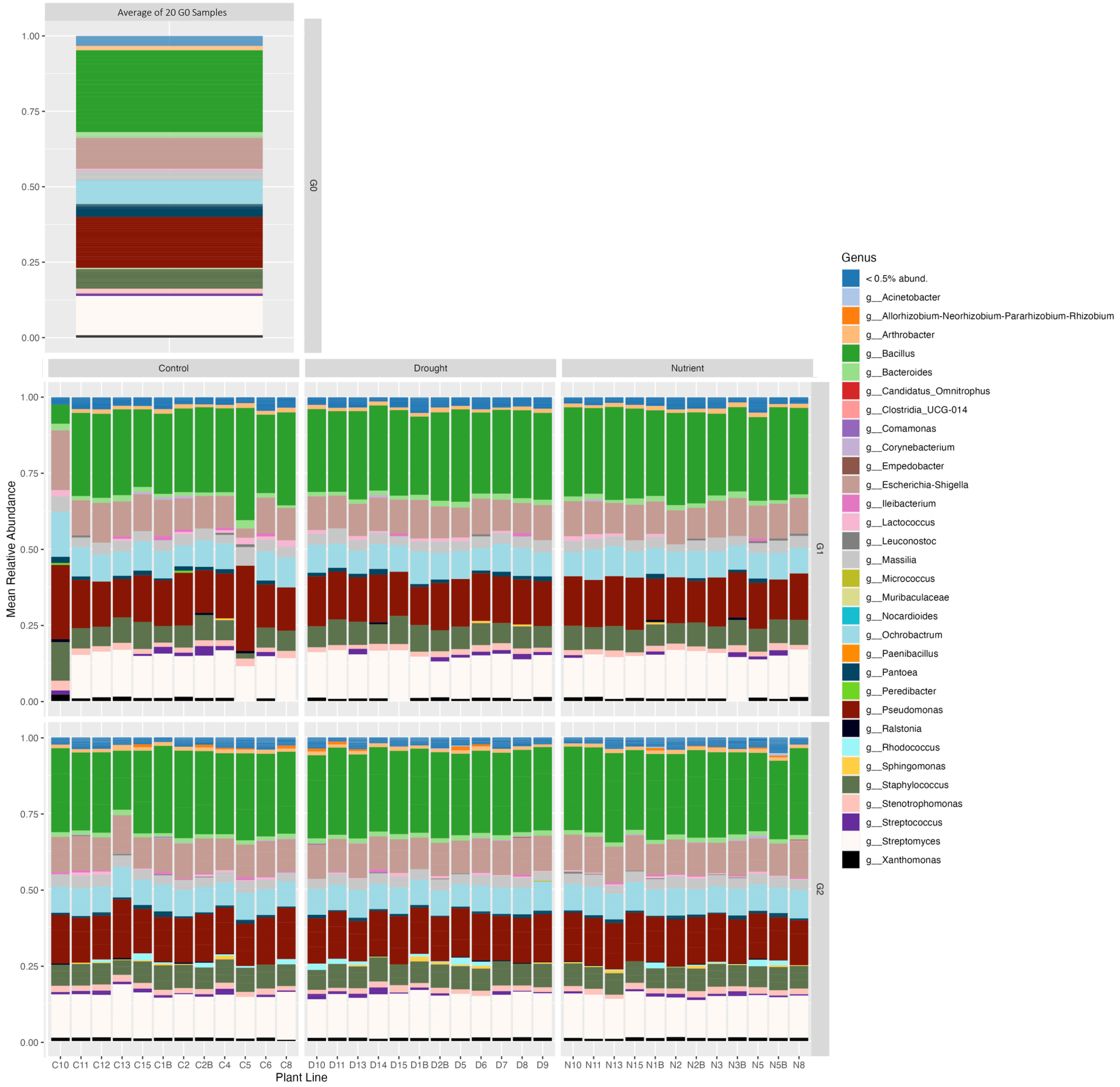

Fig. S4. Mean relative abundance of full dataset ASVs identified at the genus level across all three generations. The bar in the top row is the average of the 20 seed samples from G0. Bars in the middle row represent G1 parent samples. The bars in the bottom row represent the average of the 3 G2 offspring samples in each parent line. The “< 0.5% abund.” category comprises 263 genera less than 0.5% abundant in the dataset. “g__” indicates genus-level taxonomy.

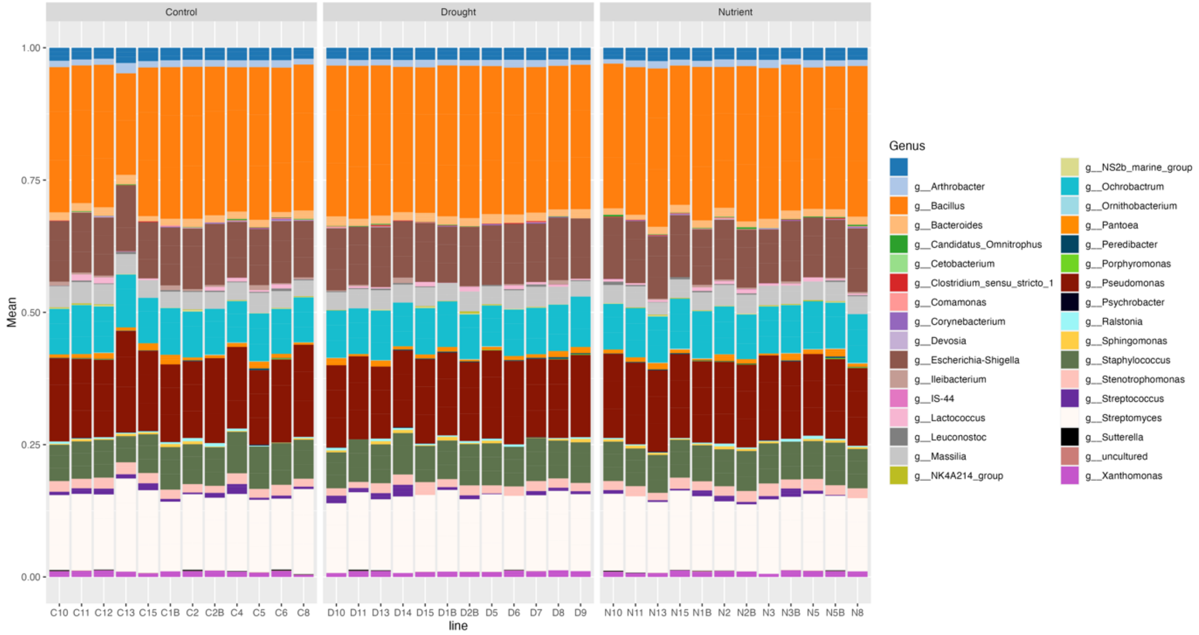

Fig. S5. Mean relative abundance of 69 prevalent overlapping ASVs detected across parent lines in G2 and identified at the genus level. All other ASVs have been removed from the dataset. The unlabeled legend color represents ASVs that were unresolved at the genus level.

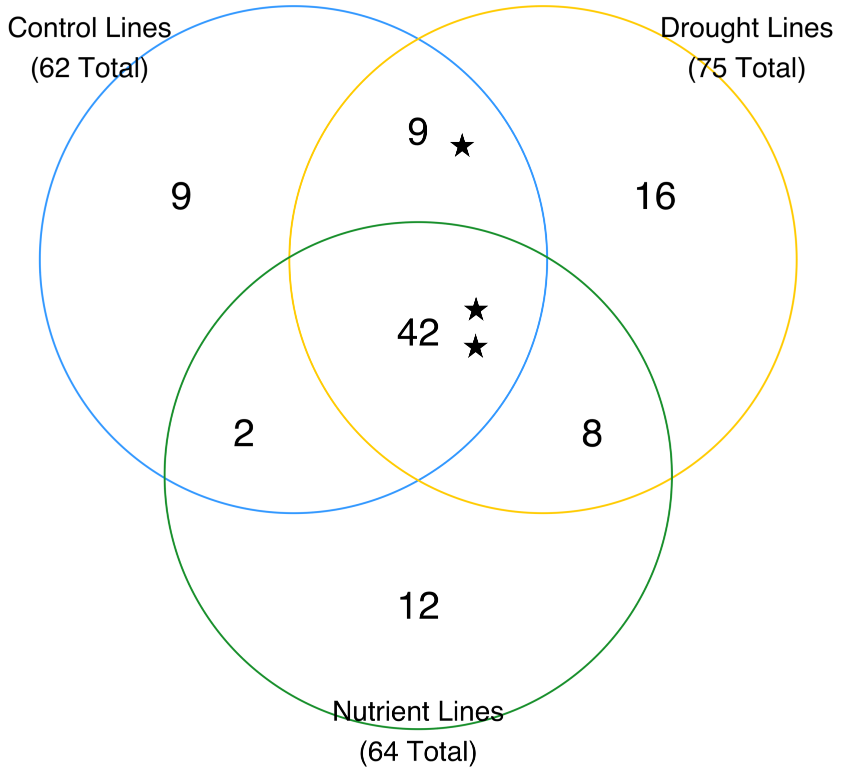

Fig. S6. Number of ASVs found overlapping between G1 and G2 within parent lines and shared between parent treatment groups. Stars indicate the presence of core seed microbiome taxa identified by Simonin et al. 2022. There are 98 total ASVs represented, of which 84 can also be found in G0.

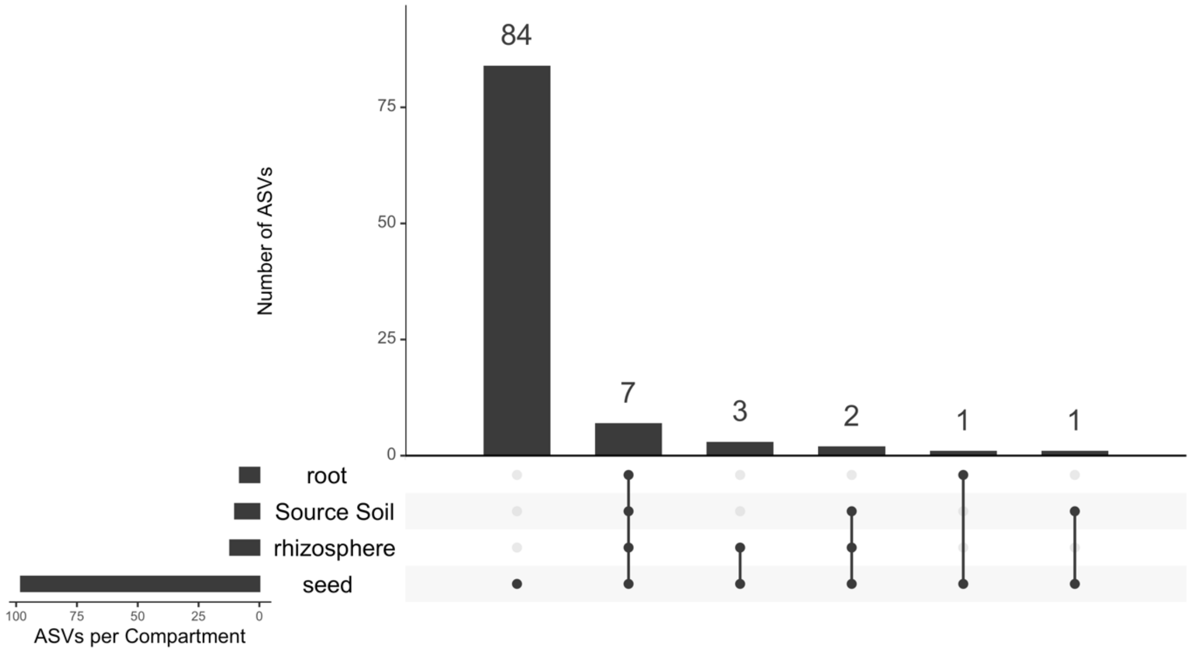

Fig. S7. Unique and overlapping ASVs between seed endophytes and bean rhizosphere soils. Of the 98 taxa identified as overlapping between the G1 and G2 seed samples within parent lines (**Table 1**), 84 were are only found in the endophytic seed samples from G1 and G2 plants, while 14 ASVs were also detected in the root, source soil, and/or rhizosphere samples across all plants.

Table S1. Excel file: Metadata table with MIMARKS compliant contextual information for each sample (e.g., plant treatment data, extraction batches).

Table S2. Statistical tests and their results to address hypotheses of differences in the alpha and beta diversity of the endophytic seed microbiome according to environmental treatment, parental line, plant generation, and their interactions.

Analysis	Statistical Test	R command/ package	Variable of Interest	Degrees of freedom	Test Statistic	P-value	
Species richness in all three generations	ANOVA	aov()/R stats	Generation	2	F = 0.446	0.641	
			G1 Treatment	2	F = 0.150	0.861	
			G1_G2 Combined Treatment	8	F = 0.393	0.923	
Species Richness in G2	ANOVA	aov()/R stats	Parent Line	35	F = 1.122	0.334	
Faith's Phylogenetic Diversity in G2	ANOVA	aov()/R stats	Parent Line	35	F = 1.123	0.332	
Beta Diversity of Weighted UniFrac distance, all three generations	PERMANOVA	adonis2()/ vegan	Generation	2	R-squared = 0.02401, F = 1.9887	0.0408	*
			Treatment	2	R-squared = 0.01617, F = 1.3397	0.1907	
Beta Diversity of Weighted UniFrac distance, G1	PERMANOVA	adonis2()/ vegan	G1 Treatment	2	R-squared = 0.05936, F = 1.0413	0.4068	
Beta Diversity of Weighted UniFrac distance, G2	PERMANOVA	adonis2()/ vegan	G1 Treatment	2	R-squared = 0.01395, F = 0.7942	0.7550	
			G2 Treatment	2	R-squared = 0.01540, F = 0.8766	0.6422	
			Parent Line	33	R-squared = 0.35582, F = 1.22276	0.0042	**
			not enough samples per group for effective post-hoc analysis with pairwise.adonis2()				
Beta diversity dispersion around spatial median, full G2 dataset	Permutational Test for Constrained Correspondence analysis	betadisper(), permutest()/ vegan	G1 Treatment	2	F = 1.4246	0.2132	
			Parent Line	35	F = 1.0388	0.4541	
Beta Diversity of Weighted UniFrac	PERMANOVA	adonis2()/ vegan	G2 Treatment	2	R-squared = 0.06343, F = 1.1863	0.2251	

distance, G2 Control Lines			Parent Line	11	R-squared = 0.34848, F = 1.1851	0.0805	
Beta Diversity of Weighted UniFrac distance, G2 Drought Lines	PERMANOVA	adonis2()/ vegan	G2 Treatment	2	R-squared = 0.04963, F = 0.9804	0.4866	
			Parent Line	11	R-squared = 0.39347, F = 1.4131	0.0032	**
Beta Diversity of Weighted UniFrac distance, G2 Nutrient Lines	PERMANOVA	adonis2()/ vegan	G2 Treatment	2	R-squared = 0.02905, F = 0.5189	0.9616	
			Parent Line	11	R-squared = 0.35515, F = 1.1534	0.2300	
Mean Relative abundance of ASVs across all generations	Kruskal-Wallis	kruskal_test() /rstatix	ASVs in 1, 2 or 3 generations across dataset	2	361.9623	2.52E -79	****
	Dunn's post-hoc with Banjamini-Hochberg correction	dunn_test()/ rstatix	1 vs 2		11.315784	1.10E -29	****
			1 vs 3		17.714023	3.27E -70	****
			2 vs 3		5.842798	5.13E -09	****
Transmission rate in G2 for 69 most common ASVs	Pearson's Chi-squared Test for Count Data	chisq.test()/ R stats	G1 Treatment	4	X-squared = 0.6705	0.9549	
			Parent Line	70	X-squared = 62.779	0.7176	

Table S3. Excel file: List of 69 ASVs from Figure 4 with taxonomic identification, core taxa membership and % identity, and 16S V4 sequence.
